# Investigating Disturbances of Oxygen Homeostasis: From Cellular Mechanisms to the Clinical Practice

**DOI:** 10.3389/fphys.2020.00947

**Published:** 2020-08-04

**Authors:** Verena Tretter, Marie-Louise Zach, Stefan Böhme, Roman Ullrich, Klaus Markstaller, Klaus Ulrich Klein

**Affiliations:** Department of Anaesthesia, General Intensive Care and Pain Therapy, Medical University Vienna, Vienna, Austria

**Keywords:** oxygen homeostasis, hypoxia, hyperoxia, intermittent hypoxia, intermittent hyperoxia/hypoxia, supplemental oxygen, oxygen therapy

## Abstract

Soon after its discovery in the 18th century, oxygen was applied as a therapeutic agent to treat severely ill patients. Lack of oxygen, commonly termed as hypoxia, is frequently encountered in different disease states and is detrimental to human life. However, at the end of the 19th century, Paul Bert and James Lorrain Smith identified what is known as oxygen toxicity. The molecular basis of this phenomenon is oxygen’s readiness to accept electrons and to form different variants of aggressive radicals that interfere with normal cell functions. The human body has evolved to maintain oxygen homeostasis by different molecular systems that are either activated in the case of oxygen under-supply, or to scavenge and to transform oxygen radicals when excess amounts are encountered. Research has provided insights into cellular mechanisms of oxygen homeostasis and is still called upon in order to better understand related diseases. Oxygen therapy is one of the prime clinical interventions, as it is life saving, readily available, easy to apply and economically affordable. However, the current state of research also implicates a reconsidering of the liberal application of oxygen causing hyperoxia. Increasing evidence from preclinical and clinical studies suggest detrimental outcomes as a consequence of liberal oxygen therapy. In this review, we summarize concepts of cellular mechanisms regarding different forms of disturbed cellular oxygen homeostasis that may help to better define safe clinical application of oxygen therapy.

## Introduction

Oxygen (dioxygen, O_2_) is mandatory to support human life from an early fetal stage onwards, as metabolism relies on its presence to produce sufficient energy efficiently in the form of adenosine triphosphate (ATP). As many acute pathological conditions are linked to hypoxemia, supplemental oxygen is frequently used as an adjunct therapy in anesthesiology, emergency and critical care medicine. However, as with most drugs, we know since the times of Paracelsus the dose makes the poison. While the organism has established elaborate mechanisms to survive hypoxic episodes, higher than current ambient oxygen conditions (21% O_2_) were rarely encountered in evolution, apart from peaks of approximately 30% O_2_ in the atmosphere during the Carboniferous period 300 million years ago and the Cretaceous period 100 million years ago (Berner et al., 2007). Excess oxygen leads to accumulation of oxygen radicals, generally known as reactive oxygen species (ROS), that have the potential to detrimentally modify proteins, lipids and nucleic acids.

In clinical practice, physicians routinely take all measures to prevent hypoxemia, accepting in many cases accidental hyperoxemia as a side effect of the therapeutic intervention. The World Health Organization (WHO) guideline in 2016 recommended, based on a meta-analysis of then current literature, that any patient who becomes anesthesized, intubated and mechanically ventilated for surgery should receive 80% inspiratory O_2_ levels during and supplemental O_2_ up to 6 h after surgery (Allegranzi et al., 2016). The recommendation was based on evidence, that such high O_2_ concentrations may exert important benefits by reducing surgical site infections (SSI), a key problem occurring after surgery worldwide. This guideline has evoked considerable criticism, as it is not known which dose of oxygen for what duration can be regarded as clinically safe (Hedenstierna et al., 2017).

Several clinical trials demonstrated that hyperoxemia may be associated with detrimental effects on mortality and morbidity in different acute conditions, including stroke, myocardial infarction (MI) and acute respiratory distress syndrome (ARDS). Further efforts in translational science, in addition to more clinical trials are needed to address this problem and to define the margins of safe oxygen application. We here discuss current experimental and clinical evidence regarding the mechanisms and consequences of dysregulated O_2_ homeostasis.

## Molecular Biology of Oxygen Sensing in Normoxia, Hypoxia and Hyperoxia

All life on earth has evolved most likely from a common ancestor around 4 billion years ago, facilitated by the chemical properties of the elements carbon and water. Early primitive organisms learned how to convert light energy into chemical energy in a process termed photosynthesis, that initially used hydrogen or hydrogen sulfide and only later, water as reducing agent. Cyanobacteria thereby produced large quantities of O_2_ which was a starting point for the evolution of the more complex living organisms. The endosymbiotic theory provides a possible explanation, how eukaryontic cells evolved: namely by symbiosis of independent prokaryotes like bacteria and archaea. The eukaryotic organelles mitochondria and chloroblasts are believed to be phylogenetically derived from Rickettsiales and Cyanobacteria, respectively. Cyanobacteria and multicellular photosynthetic eukaryotes developed via algae into higher plants, that also colonized land around 470 million years ago. Oxygen levels in the atmosphere rose and might have become a threat to other organisms, as O_2_ actively influences redox reactions, which represented a main reaction type of early life. Primitive organisms produced their fuel energy via anaerobic respiration, which maintained their life, but was rather inefficient. A major boost in evolution was the invention of using the otherwise toxic O_2_ for respiration. Bacteria capable of using O_2_ for respiration were taken up by other cells and developed into specialized cell organelles, the mitochondria, which use oxidative phosphorylation to efficiently boost the development of higher multicellular living entities. Oxygen is used to generate the universal cellular energy currency ATP in a tightly controlled cascadic pathway. Energy is released in redox reactions, where electrons flow from donors to acceptors and where O_2_ is the terminal electron acceptor, being reduced to water molecules. This electron chain, however, is not “perfect”, and some “escaping” electrons are directly transfered to O_2_, thereby generating a certain amount (1–3%) of incompletely reduced O_2_ radicals- i.e., superoxide, hydrogen peroxide and hydroxyl radical- commonly referred to as ROS.

Reactive O_2_ species are also by-products of many other cellular reactions, including oxidase/dehydrogenase enzymatic reactions of membrane-bound and cytosolic enzymes. Small amounts of ROS are used by cells as signaling molecules. Exaggerated ROS production, however, results in oxidative injury to proteins, lipids and nucleic acids (Circu and Aw, 2010). Evolution has equipped cells with antioxidative defense systems, comprised of either small molecules (vitamins, the tripeptide glutathione, redox buffer proteins – i.e., thioredoxin and peroxiredoxin) or ROS dissipating enzymes such as superoxide dismutases or peroxidases. When the anti-oxidative buffer capacity of these systems is exhausted, the cell experiences a condition of oxidative stress. It must be emphasized, that both excess as well as under-supply of O_2_ induces oxidative stress. However, the exact threshold beyond which oxidative stress harms the cells remains to be determined. Cells in different tissues and organs experience a large range of different O_2_ tensions, and accordingly, the mitochondrial electron transport chain is functional ranging from ambient O_2_ conditions (21% O_2_) down to near anoxia (approximately 0.5% O_2_). Below this limit, cellular apoptosis is initiated. From an individual cell’s view, “hypoxia” and “hyperoxia” are relative terms and are context-dependent. In this respect, “normoxia” is defined as an O_2_ level that provides optimal conditions for the cell-typical physiological processes. Any disturbance of this homeostatic balance (either up or down) results in pathological reactions.

A master regulator of cellular responses to O_2_ is the transcription factor hypoxia inducible factor (HIF)-1. In the 1990s, Gregg L. Semenza (John Hopkins School of Medicine, Baltimore, United States), together with his fellows, identified this protein, which regulates approximately 5% of the whole genome (more than 2500 target genes) (Wang and Semenza, 1995). Joint studies of the research groups of William G. Kaelin Jr. (from Dana-Faber/Harvard Cancer Center) and Sir Peter J. Ratcliffe in Oxford finally contributed to uncode the full mechanism of oxygen condition-dependent HIF-1 regulation, an achievement that was acknowledged with the shared 2019 Nobel Prize in Physiology and Medicine.

**FIGURE 1:**
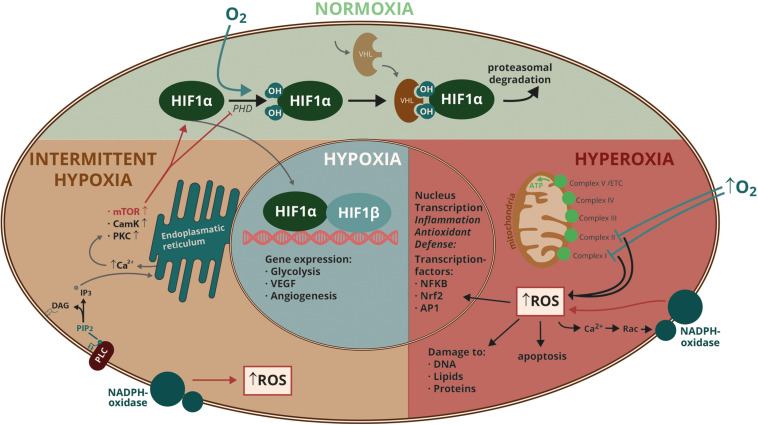
Molecular mechanisms involved in cellular responses to different oxygen conditions. Abbreviations: AP1, activator protein 1; Ca^2+^, calcium; CamK, Ca^2+^/calmodulin dependent protein kinase; DAG, diacyl glycerol; ETC, electron transport chain; HIF, hypoxia-inducible factor; IP3, inositol 1,4,5-triphosphate; mTOR, mammalian target of rapamycin; NADPH, nicotine amide adenine dinucleotide phosphate; NFKB, nuclear factor ‘kappa-light-chain-enhancer’ of activated B-cells; Nrf2, nuclear factor erythroid 2-related factor 2; O_2_, oxygen; PHD, prolyl hydroxylase domain protein; PIP2, phosphatidyl inositol (4,5)-bisphosphate; PLC, phospholipase C; PKC, protein kinase C; Rac, small GTPase; ROS, reactive oxygen species; VHL, von Hippel-Lindau protein.

Hypoxia-inducible factor-1 consists of a constitutively expressed HIF-1ß subunit and an O_2_-regulated HIF-1α subunit. Oxygen is used as a substrate by prolyl hydroxylase domain (PHD) proteins, that hydroxylate HIF-1α on proline 402 and/or 564. Proline hydroxylation allows binding to the “von Hippel-Lindau“protein (VHL) that recruits an ubiquitin ligase and facilitates proteasomal degradation of HIF-1α (Kaelin and Ratcliffe, 2008). Lack of O_2_ inhibits this reaction and therefore stabilizes the transcription factor (Figure 1). Regulation of the HIF-1 activity is complex and partly O_2_-independent via RACK-1 protein, a mechanism, that is important in cancer therapy and in the effect of the immunosuppressive drug cyclosporine A (Semenza, 2009). HIF-1 essentially regulates adaptive measures of the organism to hypoxia. Orginally identified as the transcription factor that regulates erythropoietin (EPO) transcription, HIF-1 has been shown to regulate many necessary steps involved in erythropoiesis and angiogenesis. A central role for HIF-1 in cellular metabolism is to seek the right balance between glycolysis and oxidative metabolism to achieve the maximal ATP yield under a given O_2_ condition. In this respect it is remarkable that HIF-1 can even initiate mitochondrial autophagy in order to prevent cells from death due to excess mitochondrial ROS, which is generated under both hypoxia and hyperoxia. Different levels of hypoxia are a hallmark in tumor biology. Solid tumors are frequently fast-growing cell masses with abnormal angiogenesis, where individual cells encounter a broad range of O_2_ tensions, depending on how close they are to blood vessels. In tumors HIF-1 is long-term stabilized not only by hypoxia, but frequently also over-expressed following gain-of-function mutations of oncogenes or loss-of-function mutations of tumor suppressors (Semenza, 2003).

Exposure of cells to increasing hyperoxic conditions results in an exponential release of ROS. Studies in pulmonary capillary endothelial cells, using DCF (2′,7′-dichlorofluorescein) fluorescence imaging and specific inhibitors of mitochondrial complexes or nicotinamide adenine dinucleotide phosphate (NAD(P)H) oxidase, revealed an early phase, where ROS was predominately released from the mitochondrial electron transport chain as a consequence of complex I and II inhibition, and a late phase with more ROS being released by NAD(P)H oxidase. Mitochondrial ROS initiates a calcium (Ca^2+^) signal, that translocates Rac1 (a small GTPase) to the plasma membrane, where it activates NAD(P)H oxidase (Brueckl et al., 2006) (Figure 1). This cascadic release of ROS might indicate, that short-term hyperoxia is capable of activating endothelial cells, but is not necessarily toxic. The time-shift between the two steps observed in above mentioned experiment was 30 min. Longer exposure times, however, lead to massive ROS amplification, that overwhelms the cell’s defense systems and lead to irreversible damage. Hyperoxia further disturbs the necessary balance of mitochondrial fusion and fission and favors mitochondrial fragmentation, a phenomenon that is similarily observed in different pathological conditions such as diabetes, artherosclerosis and pulmonary artery hypertension (Ma et al., 2018). The increase of ROS during hyperoxia affects a large number of intracellular signal transduction proteins, including protein kinases, transcription factors, channels, receptors and members of the apoptosis pathway (Gore et al., 2010). Central to the molecular responses to hyperoxia are the redox-activated transcription factors nuclear factor, erythroid 2 related factor 2 (Nrf2), nuclear factor kappa B (NF-KB) and activator protein-1 (AP-1) (Wright and Dennery, 2009). Nrf2 regulates the expression of ROS-detoxifying enzymes such as glutathione S-transferase, superoxide dismutase, catalase, NAD(P)H quinone oxidoreductase and the oxidative stress response protein heme oxygenase (HO-1) via DNA sequences called antioxidant response elements (ARE). Nrf2 was shown to be an important protector against hyperoxic lung injury in linkage analysis (Cho et al., 2002). Detailed bioinfomatic and genetic studies on humans as well as transgenic animal models revealed the lung-protective effect of Nrf2 (Cho and Kleeberger, 2010). AP-1 is composed of fos and jun proteins and controls cell proliferation and death in response to stimuli like hyperoxia. Hyperoxia-induced cell death is enhanced when AP-1 activation is blocked (Li et al., 2003). Activated AP-1 also targets the IL-8 promoter, providing a mechanistic link to the inflammatory response in hyperoxia (Joseph et al., 2008; Wu et al., 2016a). NF-KB is a dimeric transcription factor that targets genes involved in the regulation of apoptosis, inflammation and oxidative stress. Activation of NF-KB has a “canonical” and an “atypical” pathway, with the latter being especially important in the response to hyperoxia (Wright et al., 2009). Hyperoxic NF-KB activation is cell-type specific, and so is the response of different cell types in the lung to hyperoxia. From 40 different cell types in the lung, endothelial cells are especially sensitive to hyperoxic injury, while type II epithelial cells are resistant (Crapo et al., 1980). Endothelial dysfunction and destruction as well as inflammation are hallmarks of phase II in pulmonary oxygen toxicity (as discussed later in this review). It has been discussed, that endothelium’s susceptibility to hyperoxic lung injury is caused by its high metabolic activity and its close contact to a large number of “active” substances from the circulation (Kallet and Matthay, 2013). Endothelial metabolism has been shown to be affected by high oxygen concentrations with a subsequent dysregulation of vasoactive substances. Hyperoxic lung injury is enhanced by an increased metabolism in these cells, probably also by an elevated oxidative phosphorylation and concomitant ROS generation. The endothelial nitric oxide synthase (eNOS) generates large amounts of nitric oxide (NO) and both, the enzyme and its reaction product are sources of aggressive ROS and radical nitrogen species under hyperoxic conditions. Also, the antioxidative defense is cell-type specific and might make the endothelium especially vulnerable. Phase III in pulmonary O_2_ toxicity constitutes a pro-coagulative state and the formation of microthrombi, which is supported by the activation of mediators like plasminogen activator inhibitor (PAI)-1 in lung epithelial cells by inflammatory cytokines induced by NF-KB (Jacobson and Birukov, 2009). Further, NF-KB has a differentiated and sophisticated role in embryonal development, a major reason why it has not been developed as a general drug target for treatments. However, in the case of patients with acute lung injury, that require mechanical ventilation with high levels of O_2_, a combination of the anticoagulant protein C and statins, which inhibit NF-KB and downregulate PAI-1 have been proposed to be tested in clinical trials (Liu et al., 2009). Several other factors contribute to the complex response of the cell to hyperoxia, i.e., STAT (signal transducers and activators of transcription protein)-subtypes, CEBP (ccat/enhancer binding protein), c-myc, Fra-1, junB and c-Fos. To better understand the tissues’ responses to hyperoxia, several recent studies investigated proteomic alterations in response to various degrees of hyperoxia (Konsavage et al., 2013; Hinkelbein et al., 2015; Hafner et al., 2017b). The complexity of the cellular proteome as well as the use of different experimental and analytical protocols necessarily leads to different results and conclusions in these studies. However, the analysis of the proteome adds significantly to the understanding of ongoing mechanisms, as gene expression analysis not necessarily directly reflects steady-state protein levels. Further proteomic investigations using specific cell types in culture and also further animal experiments are indicated to obtain a detailed understanding of hyperoxic systems biology.

Alternating O_2_ conditions, even with oscillating patterns, are observed in many clinical conditions and diseases - e.g., tumors, asthma, sickle cell disease, smoking, obstructive sleep apnea syndrome (OSAS) and cyclic recruitment and derecuitment of atelectasis during mechanical ventilation in patients with acute respiratory distress syndrome (ARDS). These conditions differ importantly in extent of frequencies of oscillations, O_2_ ranges and O_2_ amplitudes – most likely causing different cellular responses (Almendros et al., 2014). For example, pulmonary O_2_ oscillations caused by cyclic recruitment and derecruitment during ARDS are not only local phenomenons within the lungs on an alveolar level, but have been shown to be transmitted as arterial O_2_ oscillations to remote tissues and organs, including the brain (Klein et al., 2013) and the kidneys (Thomas et al., 2017).

Baumgardner, Klein, Markstaller and others have hypothesized that these O_2_ oscillations might represent an independent pathomechanism in ventilator-induced lung injury that might aggravate biotrauma (Boehme et al., 2019). Cell culture studies confirmed that O_2_ oscillations in the hyperoxic/hypoxic range result in specific pathway activations that are different from constant hypoxic/hyperoxic conditions (Nanduri and Nanduri, 2007; Wu et al., 2016a, b; Hafner et al., 2017a; Wohlrab et al., 2018). HIF-1α has been shown to be activated faster by intermittent hypoxia than by continous hypoxia, though through different mechanisms (Nanduri and Nanduri, 2007). Intermittent hypoxia induces ROS production by NADPH oxidase, activating phospholipase (PLC)γ, which generates inositol 1,4,5-triphosphate (IP3) and diacylglycerol, thereby mobilizing intracellular Ca^2+^. Calcium activates calcium-calmodulin kinase (CamK), protein kinase C and finally mTOR, which facilitates HIF-1α synthesis and inhibits PHD2-dependent degradation (Figure 1). Intermittent hypoxia, as occuring in OSAS, is believed to activate the carotid body, which normally detects hypoxia, leading to an increased sympathetic tone and systemic hypertension. Besides differences in HIF-1α activation, O_2_ oscillations also affect more signaling pathways including other transcription factors, kinases and phosphatases. In particular, intermittent hypoxia has been shown to have a larger impact on the activation of the immediate early gene c-fos than hypoxia itself, resulting in an increased AP-1 transcriptional activity (Yuan et al., 2004). NF-KB is another transcriptional activator that is enhanced by intermittent hypoxia (Greenberg et al., 2006). Sustained and intermittent hypoxia also have divergent effects on activation of signaling pathways as exemplified by the differential activation of MAPkinases, PKC and CamKII (Yuan et al., 2005). Alterations in the activity of these pathways affect cellular differentiation, proliferation as well as inflammation and apoptosis.

Intermittent hyperoxia has been less investigated as compared to intermittent hypoxia, although it frequently occurs in the clinical setting, mainly through supplemental O_2_ via facemask, or even in neonates ventilated with room air. Combinations of intermittent hypoxia and intermittent hyperoxia have been shown to blunt inflammatory responses (Hafner et al., 2017b) as well as physiological reactions (Bavis et al., 2019). Whether this is attributable to physiologic antagonism or specific and divergent signaling mechanisms remains to be investigated in more detail. The study of Hafner et al. directly compares cardiac cell responses after different time points in response to normoxia, hypoxia, hyperoxia and intermittent hypoxia/hyperoxia. Comparing the release of cytokines under these conditions reveals frequently an intermediate response of intermittent hypoxia/hyperoxia. Interleukin-8 release is much increased under intermittent hypoxia/hyperoxia, which might be a result of a strong activation of NF-KB under chronic oscillations. Quantitative proteomics of cell extracts revealed frequently, but not in all cases an opposite tendency in the level of some signaling or structural proteins, which implicates the complexity of alterations of responses.

## Physiology of Oxygen Sensing in Normoxia, Hypoxia and Hyperoxia

The concentration gradient of partial O_2_ pressure within supplying blood vessels and surrounding tissues is the main determinant and the driving force of O_2_ supply by diffusion through membranes to the tissues. In addition to the pressure gradient, effective levels of dissolved O_2_ are altered by barometric pressure, temperature, humidity, physiological conditions and all factors acting on the dissociation curve of O_2_ from hemoglobin (Ortiz-Prado et al., 2019). The inspired partial pressure of O_2_ in the upper airways is reduced from 159 mmHg present in room air to 100 mmHg in the alveolar compartment (pAO_2_), mostly due to the addition of water vapor, the effect of dead space ventilation and the mixing of inspired gas with expired gas. As the pAO_2_ cannot directly be measured, it needs to be calculated from the alveolar gas equation. Rapid gas exchange at the alveolar-capillary membrane results in an arterial partial pressure (paO_2_) that is almost equal to the alveolar partial pressure of O_2_ (pAO_2_ = 75–100 mmHg) under healthy conditions. This arterial partial pressure remains unchanged for the first decades of life and then slowly declines with age due to alterations in the ventilation-perfusion matching within the lungs. In smokers or patients with lung diseases, this decline occurs more rapidly and is more pronounced. The average partial pressure of O_2_ in tissues is designated ptO_2_. Tissue partial pressure of O_2_ is dependent on blood flow, oxygen availability and consumption. Intracellular partial pressure of O_2_ (ipO_2_) determines availability of oxygen to mitochondria and energy production.

Homeostasis with regard to O_2_ supply is maintained by several O_2_ sensing systems (Weir et al., 2005). A decrease in the arterial partial ressure of O_2_ is sensed by the glomus cells of the chemoreceptor organ “carotid body” (CB). This peripheral O_2_ sensor is a small para-ganglion at the bifurcation of bilateral Arteriae carotis communis each into the Arteriae carotis interna and externa and was discovered by Jean-Francois and Corneille Heymans in the 1920s. A drop in the partial pressure of O_2_ (hypoxemia) or pH (acidosis) as well as a raise of the partial pressure of carbon dioxide (hypercapnia) lead to a depolarization of the cell membrane, a block of potassium channels (TASK and K_V_ channels) and opening of voltage-dependent L-type calcium channels. The elevated intracellular calcium initiates the exocytosis of neurotransmitter-laden vesicles that activate via their cargo afferent nerve fibers (Renshaw and Nikinmaa, 2007). Afferent axons from the CB send signals to the Nucleus tractus solidarius (NTS) in the caudal medulla. Projections then reach the respiratory neuronal network and brainstem autonomic sympathetic nuclei (rostral ventrolateral medulla, RVLM) From there, the respiratory rate is regulated and the adrenal medulla is stimulated to increase catecholamine release (adrenaline, noradrenaline) leading to an increased heart rate and blood pressure (Semenza and Prabhakar, 2018). Enhanced CB input can lead to an overactivation of the sympathetic nervous system resulting in pathological developments like hypertension, sleep apnea and the metabolic syndrome. In some cases, ablation of the CB is considered as a therapy to treat these diseases (Iturriaga et al., 2016). The brain lies downstream of the arterial respiratory chemoreceptors and as animal experiments indicate, possesses its own central O_2_ response system. Key players seem to be astrocytes, where hypoxia induces an inhibition of the mitochondrial respiratory chain resulting in an increase of ROS. Further signaling activates phospholipase C and calcium release from internal stores. The astroglial calcium signals are highly sophisticated and induce multiple downstream effects. They can be transmitted to adjacent glia cells via connexins and can induce the exocytosis of messenger-laden vesicles. An important messenger in the response to hypoxia is ATP, which binds to purinergic receptors also found on neurons, that generate the respiratory rhythm (pre-Bötzinger Complex neurons) and sympatoexcitatory neurons of the ventrolateral medulla oblongata (Gourine and Funk, 2017; Ramirez et al., 2018).

The respiratory center consists of four respiratory groups: dorsal and ventral respiratory group (medulla) and pneumotaxic and apneustic center (pons). From there the diaphragm is activated to allow air moving in and out of the lungs to exchange O_2_ for the exhaled CO_2_. Depression of the central respiratory center (due to diseases or drugs) can lead to a failure in ventilation and respiratory acidosis due to an accumulation of the acidic CO_2_. Respiratory acidosis is corrected to some extent by the cellular and plasma buffers and secondary renal compensation. In 1977, Lauweryns described in the airway mucosa neuroepithelial bodies (NEB), which are widely distributed in lungs, and are innervated clusters of amine (serotonin) and peptide producing cells containing O_2_ sensitive K+ channels (Lauweryns et al., 1977; Cutz et al., 2013). As they are similar to CB and as they are especially numerous in the fetal and neonatal lungs, when the CB are not yet fully developed, it has been proposed, that they serve a similar purpose early in development.

Low O_2_ tensions in the alveolar space induce a lung-specfic phenomenon called “*hypoxic pulmonary vasoconstriction*” (HPV; Euler-Liljestrand mechanism), where smooth muscle cells in the pulmonary arteries contract in response to hypoxia in order to redirect the blood-flow to well-ventilated lung areas, and to improve the ventilation/perfusion ratio. Although the exact mechanism leading to O_2_ sensing by the precapillary smooth muscle cells remains to be elucidated, involved signaling pathways may include activation of voltage-gated potassium channels, calcium channels and transient receptor potential channels (Archer et al., 1998; Dunham-Snary et al., 2017). This hypoxia-induced pulmonary vasoconstriction causes pulmonary hypertension when breathing in low inspired O_2_ tension situations as encountered in exposure to high altitudes. Supplemental O_2_ under such circumstances leads to rapid and efficient relief of both pulmonary hypertension and tissue edema (Aragones et al., 2009).

Hypoxia also has an impact on the acid-base equilibrium in the body and depending on the condition might induce either alkalosis or acidosis (Swenson, 2016). Hyperventilation or exposure to high altitude can result in alkalosis with reduced sympathetic tone, blunted hypoxic pulmonary vasoconstriction and increased hemoglobin O_2_ affinity. Severe hypoxia or ischemia frequently results in metabolic and hypercapnic acidosis (due to lactate formation and CO_2_ accumulation). Hypoxia and the Warburg effect (aerobic glycolysis for massive proliferation) play a major role in the rapid growth of tumors, where HIF-1 signaling regulates many genes involved in acid-base heomeostasis.

When an individual breaths hyperoxic gas mixtures, the partial pressure of arterial O_2_ will increase. The physiological limits can be expanded by using higher pressures (hyperbaric hyperoxic O_2_), which is clinically used to treat conditions like traumas. The net effect is a steeper intracellular O_2_ gradient promoting better diffusion from the capillaries to the tissues. After an initial transient decrease in ventilation a paradoxical increase of the respiratory response follows (hyperoxic hyperventilation), that is centrally regulated. High concentrations of oxygen further completely saturate hemoglobin, while at the same time the affinity for CO_2_ is decreased (Haldane effect). The resulting acidification induces an enhancement of CO_2_ removal via hyperventilation (Brugniaux et al., 2018).

Harmful effects of O_2_ mainly depend on concentration and pressure. Oxygen toxicity under normobaric conditions (“normobaric hyperoxia”) is mainly manifested as oxidative damage to the respiratory epithelium of the tracheobronchial tree with symptoms like painful breathing, chest pain and dyspnea and depends on exposure time and concentration. A benchmark is a more than 24h exposure to a fraction of inspiratory O_2_ (FiO_2_) > 0.6. A clinical read-out for occurring O_2_ toxicity is a measureable reduction in vital capacity of lung function, followed by tachypnea and increasing hypoxemia leading to respiratory failure. Horncastle and Lumb (2019) describe four stages in pulmonary toxicity: 1. increase in ROS and reduction of antioxidative defense; 2. inflammation, endothelial and surfactant damage leading to hyperpermeability and edema 3. activation of coagulation, formation of microthrombi; 4. collagen deposition and fibrosis. Clinical diagnosis is difficult as other co-existing conditions might produce similar symptoms. Hyperbaric hyperoxia can result in central nervous system toxicity with symptoms like nausea, headache, dizziness, visual and mental disturbances and is frequently encountered by oceanic divers. Apart from pulmonary function tests a novel approach to detect oxygen toxicity is the analysis of exhaled breath for volatile organic compounds (Wingelaar et al., 2019).

## Assessing Oxygen Sensing *In Vitro* and *In Vivo*

The most widely used experimental approach to investigate molecular responses of living cells to altered gas conditions are *in vitro* cell cultures. The implementation of such experiments initially proved very cumbersome due to low solubility and slow diffusion of gases in culture media (Place et al., 2017). A major criticism arises from the fact that many cell culture experiments use ambient O_2_ levels of 18–21% O_2_ as control conditions, a level of “normoxia“, that differs importantly from the O_2_ levels encountered by cells under physiological conditions in their natural environment (1–13% O_2_; “physioxia” or tissue normoxia). Cells are usually cultivated for days to weeks or even longer under such “relative” hyperoxic conditions and might have adapted their metabolism to these new conditions. However, well-defined cell cultures are still the starting point, when the activation of signal transduction pathways is of central interest in the investigation. Here, technical limitations with regard to fast cellular delivery of gases have been overcome in the recent years (Pavlacky and Polak, 2020). Several systems using cell culture dishes with gas-permeable membranes have been introduced. These membrane-based systems offer the advantage of direct delivery of gases to the cultured cell layer without the need for slow equilibration of the culture medium (Wu et al., 2016b; Farre et al., 2018). Depending on the material of the gas-permeable membrane, very fast transition times can be achieved (below 0.5 s for polydimethylsiloxane (PDMS) membranes on silicone basis). This is especially important when investigating high frequency oscillations (up to 300 cycles/h as sometimes used in mechanical ventilation). In addition, these gas-permeable membrane-based systems omit the need for repetitive exchange of equilibrated medium to induce changes in O_2_ levels, thereby avoiding exposure of the cells to shear stress (Tsapikouni et al., 2012). Shear stress is also an issue in perfusion-based systems, but has been minimized in setups using microfluidic chips (Pavlacky and Polak, 2020). To separate the differential contribution of shear stress and strain on ventilator induced lung injury (VILI) as compared to O_2_ toxicity, *in vitro* model systems exist that are capable of simulating mechanical stress on cultured cells. Various systems have been described and many of them were custom-built. Cell cultures are cultivated in special chambers and are then exposed to different types of cyclic stretch and shear stress followed by analysis of gene expression, visualization of cytoskeletal rearrangements or calcium (Ca)^2+^ fluxes. Using such an *in vitro* VILI model, it has been shown that VILI, in combination with hyperoxia, changes cell membrane properties (enhances stiffness) and leads to rearrangement of the cytoskeleton. The concomitant increase in resistance to deformation exposes cells to mechanical strain and stress-induced injury (Roan et al., 2012). The premium class of *in vitro* cell culture systems are three-dimensional (3D) cell cultures, especially when multiple cell types are included. A special challenge in these cultures is the formation of O_2_ gradients, which need to be monitored with microelectrodes or other O_2_ reporters (Pavlacky and Polak, 2020).

The majority of animal models for studies of hyperoxia and hypoxia have been performed in rodents, mainly rats or mice. Beside their ease in breeding, handling, storage, cost, etc, these animal models offer the unique opportunity to manipulate the genetic background of the respective strain, which has provided the path to specific deletion or overexpression of single genes. In addition, the long history and the vast experience with these models provide a solid background for the experimental design of studies. For example, newborn rodents exposed to hyperoxia provide a simple model for BPD, as their lung development is not finalized upon birth, but at a stage similar to an extremely prematurely born human (saccular stage of lung development). A recent study characterized a rat model of BPD in detail by exposing neonatal rats to a FiO_2_ of 0.6–1.0 from day of life 1–19 (Greco et al., 2019). These hyperoxic challenged neonatal rats developed severe growth restriction, rarefaction of pulmonary vessels, alveolar simplification, increased a-SMA (smooth muscle actin) content in vessels (indicating lung fibrosis due to differentiation of lung fibroblasts into myofibroblasts) and right heart hypertrophy.

A large number of experiments have been performed using rat or mouse models of VILI and HALI, or combinations of both, indicating that high O_2_ concentrations aggravate VILI symptoms. Effects are even worse in older animals, probably due to increasing pro-oxidant developments and loss of antioxidant defense mechanisms during aging (Andrade et al., 2014). As the basal metabolic rate is linearly inversely related to animal size, small animals live and exercise on an elevated level of aerobic metabolism, being more sensitive to phenomenona like VILI and HALI. Therefore, translation of experimental results is not necessarily straightforward.

### Measuring Oxygen

One of the first available methods to measure blood oxygenation was spectroscopy based on the observation that oxygenated and de-oxygenated hemoglobin have different absorption curves over the spectrum of wavelengths. Initial inventions of oxygen sensitive electrodes in the early 1950s using a polarized platinum cathode and a nonpolarizable silver anode were advanced by Leland Clark to measure partial pressures of O_2_ in blood gas analysis (“Clarke-type Electrodes“) (Clark et al., 1953), and were further developed in the following years. A major disadvantage of Clark-type electrodes is the fact, that they consume O_2_; therefore, measuring very low levels of O_2_ can be biased.

In the 1970s, the Japanese bioengineer Aoyagi developed a method which is now known as pulse oximetry, measuring O_2_ saturation (SO_2_) of the blood (Aoyagi and Miyasaka, 2002). In this method, two wavelengths of light are passed through a thin body part, and changes in the absorption by the pulsing arterial blood are measured. In the 1980s, Wilson et al. developed platinum and palladium-based probes that, when excited in the UV range, exhibit an O_2_ sensitive decay of phosphorescence emission. Later ruthenium probes with fluorescence quenching were used (Boehme et al., 2013).

While under physiological conditions small alterations (e.g., 10–20 mmHg) in paO_2_ can occur in patients with healthy lungs, significant paO_2_ oscillations can be evident due to injured lungs (e.g., in ARDS) as a consequence of cyclic changes in shunt fraction and cyclic opening and collapse of alveoli. Formenti et al. developed ultrafast fiberoptic O_2_ sensors with a fast response time that can be used to detect rapid variations in paO_2_, and can potentially be used in humans (Formenti et al., 2015, 2017). These probes can be used to visualize within-breath oscillations of paO_2_. Current research proposes to use the information gained from dynamic monitoring to tailor ventilator settings according to the individual patients’ conditions (Formenti and Farmery, 2017).

Positron emission tomography (PET), magnetic resonance imaging (MRI) and electron spin resonance (ESR/EPR) are more sophisticated imaging methods used to visualize hypoxic regions *in vivo.* These methods are used for diagnosis in humans as well as with adaptations to research animals also in pre-clinical research (Mason et al., 2010). PET uses positron-emitting isotopes (^18^F, ^64^Cu) for instance of imidazole derivates that diffuse into cells, where they are reduced and trapped. Under normoxia the tracer is reoxidized and leaves the cells. For example, ^15^O-oxygen labeled gas tracers are used to evaluate cerebral O_2_ metabolism in humans. The method has been also adapted to research animals (rats) with some modifications to overcome technical challenges. For a better application of the tracer gas and intravenous administration method has been developed. Rat blood is collected and red blood cells are labeled with ^15^O-oxygen gas using an artificial lung (Magata et al., 2003). The labeled blood is then injected into the animal for the experiment. Small rodents are preferred disease models to study conditions like neurodegenerative diseases and cerebral ischemia. PET is used to assess parameters like cerebral metabolic rate of oxygen (CMRO_2_), cerebral blood flow (CBF), cerebral blood volume (CBV) and oxygen extraction fraction (OEF).

Magnetic resonance imaging (MRI) methods are frequently used to assess the degree of hypoxia in tumors in pre-clinical research and, if suitable for humans, also in clinical diagnostics. MR signals arise from the transition of dipoles (hydrogen nuclei; ^1^H) between different energy states. The subject is posed in a static magnetic field (with field strength measured in Tesla), where the dipoles align according to the magnetic field. Radio frequency pulses are used for the transition of the dipoles to a higher energy level at the resonance frequency. Based on this principle individual body structures, perfusion or even functional parameters can be displayed. The ^1^H signals can be modified with the help of contrast agents, which are metal-based, ^19^F or use the BOLD contrast mechanism.

Blood-oxygenation level-dependent magnetic resonance imaging (BOLD-MRI) can visualize stimulus-dependent activations of brain activity and relies on changes in the magnetic properties of oxygenated and deoxygenated hemoglobin. Oxygen consumption increases due to increased brain activity will lead to a change in cerebral blood flow with a concomitant reduction of paramagnetic deoxyhemoglobin. The method may yield procedure-related biased results under general anesthesia (Logothetis and Wandell, 2004). BOLD signals are used to provide insight into neuronal activity associated with different tasks. The responses relate to changes in cerebral blood volume, blood flow and O_2_ consumption with mutlifactorial influencing factors (neuronal activity, metabolism, type of neuronal circuit, cell type and many others). Therefore, data interpretation with respect to information processing in the brain is still highly challenging.

In electron paramagnetic (spin) resonance (EPR/ESR) oximetry, the absorption of energy by unpaired electrons in the sample at radio- or microwave frequencies is measured, and provides information about the magnetic environment (Williams et al., 2010). Free radicals containing unpaired electrons are exposed to microwaves in a changing magnetic field until resonance occurs. In research ESR is used *in vitro* to understand the role of free radicals such as ROS and nitric oxide (NO). Non-invasive *in vivo* measurements are feasible, but technically more challenging due to sensitivity and influence of radical scavengers. For these measurements low-frequency ESR spectroscopy has been developed for measurements in whole research animals (Ohya-Nishiguchi and Packer, 1995). A reporter probe is injected into the tissue before the measurement. This method is used to monitor tissue oxygenation during disease and therapy.

## Pathophysiology of Oxygen Sensing Dysregulation: Hypoxia, Hyperoxia and Intermittent Hypoxia

**FIGURE 2:**
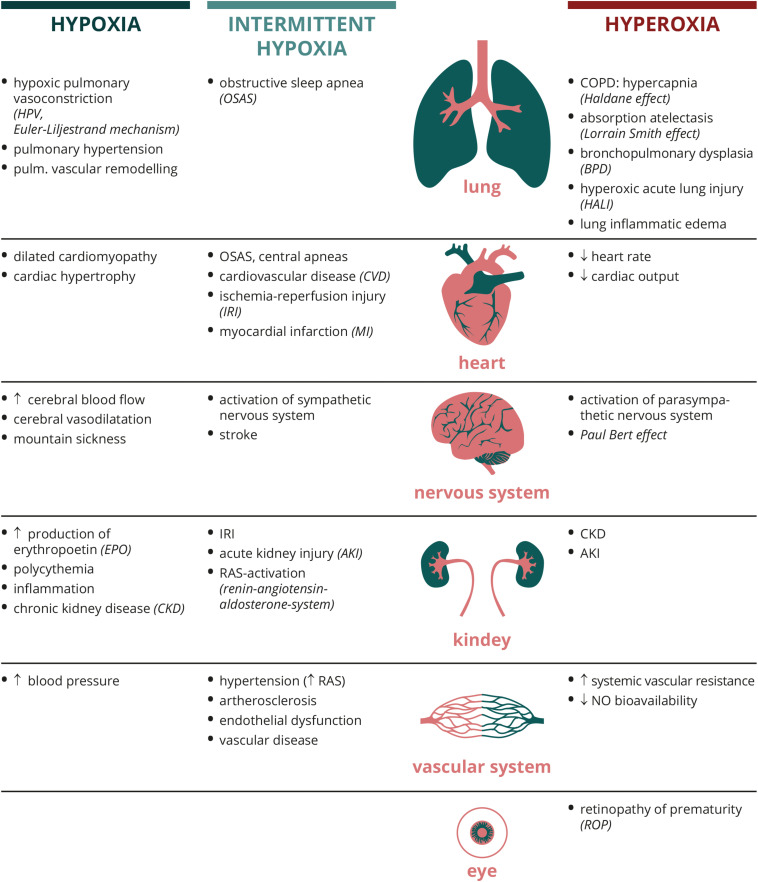
Organ-specific physiological and pathological responses to different oxygen conditions. Abbreviations: AKI, acute kidney injury; BPD, bronchopulmonary dysplasia; COPD, chronic obstructive pulmonary disease; CKD, chronic kidney disease; CVD, cardiovascular disease; EPO, erythropoetin; HALI, hyperoxic acute lung injury; HF, heart failure; HPV, hypoxic pulmonary vasoconstriction; IRI, ischemia reperfusion injury; MI, myocardial infarction; NO, nitric oxide; OSAS, obstructive sleep apnea syndrome; RAS, renin-angiotensin-aldosterone-system.

The essential role of oxygen for physiologic cell metabolism at rest and under stress implicates that any disturbance in the supply-demand ratio can lead to significant pathophysiologic reactions. Figure 2 depicts examples; how different organ systems respond to dysregulated O_2_ conditions.

### Lungs

In order to provide efficient oxygenation of the blood the lung responds to hypoxic conditions with the physiological response of *hypoxic pulmonary vasoconstriction* with the purpose to redirect the blood flow from lesser to more oxygenated areas of the lung. After phases of acute hypoxia, arteries can reversibly dilate. Chronic general hypoxic conditions (as in high altitude living or conditions like chronic obstructive pulmonary disease), however, lead to widespread vasoconstriction thereby increasing blood pressure in the lung with a concomitant exuberant stress on the heart. This hypoxic pulmonary hypertension is accompanied by a remodeling of the vessels, especially the pulmonary artery (Tuder, 2018). This includes enhanced proliferation and dedifferention of vascular smooth muscle cells leading to a loss of contractility, hyperplasia and hypertrophy, extracellular matrix deposition, endothelial cell dysfunctions and muscularisation of normally non-muscular arteries. Many of these alterations are mediated by the cellular oxygen sensors of the hypoxia-inducible factor (HIF) family.

Intermittent hypoxia with regular reoxygenation events activates different mechanisms. A classical disease associated with intermittent hypoxia is *Obstructive sleep apnea syndrome (OSAS).* OSAS is a breathing disorder occurring as a consequence of cyclically collapsed upper airways during sleep with a high prevalence, especially among obese patients. The resulting cycles of hypoxia-reoxygenation (intermittent hypoxia) generate large amounts of ROS, activate HIF-1α and de-activate its antagonist HIF-2a. Consequently, patients suffer from hypertension and are proned to develop secondary organ damage and diabetes.

Supra-normal paO_2_ levels (e.g., caused by applying high FiO_2_ levels) have an impact upon pulmonary, vascular, metabolic and cerebral effects (Hafner et al., 2015). A rise in alveolar and mixed venous pO_2_ inhibits hypoxic pulmonary vasoconstriction and leads to “*absorption atelectasis*” with increased right-to-left shunt due to low ventilation/perfusion ratios (V_A_/Q) (Lorrain-Smith effect). This is not observed under hyperbaric conditions, and is partially prevented by continuous positive airway pressure breathing or a higher positive end-expiratory pressure (PEEP). However, longer periods of high oxygen breathing are associated with the danger of oxygen toxicity, which presents itself as severe pulmonary inflammation and edema. It is worthy to note that when periods of breathing 100% O_2_ are interspersed with short periods of normal air breathing, O_2_ toxicity may be attenuated (Hendricks et al., 1977). We have evidence from *in vitro* experiments on cardiomyocytes, vascular endothelial cells and pulmonary endothelial cells that intermittent hyperoxia/hypoxia (hyperoxia interspersed with hypoxic periods) over a longer time (72 h) results in significantly lower amounts of pro-inflammatory cytokine release (Hafner et al., 2017b).

#### Hyperoxic Acute Lung Injury (HALI)

Patients with respiratory failure are mechanically ventilated using supraphysiological concentrations of O_2_. This intervention is frequently life-saving in the short-term, but prolonged exposure to high O_2_ breathing gas produces large amounts of ROS from mitochondria and NADPH oxidase NOX1. These radicals are aggressive *per se*, thereby oxydizing proteins, lipids and nucleic acids, but also engage in cellular signaling. Downstream of NOX1 MAPkinases ERK1/2, JNK and p38 are activated (Dias-Freitas et al., 2016). Cell death is initiated by activation of caspases 3 and 9, and finally NF-KB activation results in release of pro-inflammatory cytokines such as interleukins IL-1ß, IL-6, IL-8, tumor necrosis factor (TNF)α and macrophage inflammatory protein (MIP-)2. Extracellular HMGB1, a pro-inflammatory damage (danger) associated molecular pattern (DAMP), is increased, promoting ALI progression. The HMGB1 receptor RAGE (receptor for advanced glycation endproducts) plays a major role in normal lung development and functionality. During hyperoxia, RAGE levels are increased, mediating lung inflammation. Calfee et al. showed that lung protective ventilation (V_T_ = 6 ml/kg) can lower RAGE levels (Calfee et al., 2008). Destruction of the alveolar-capillary barrier is due to endothelial and epithelial cell apoptosis, and changes in the expression of Na^+^ channels and the Na/K ATPase of alveolar type-II (ATII) cells disturb the fluid balance and ultimately lead to edema. Chemokines attract neutrophils and macrophages that accumulate in the interstitium and air spaces. Activation of phagocyte NOX2 further increases ROS levels and leads to cell death.

#### Broncho-Pulmonary Dysplasia (BPD)

Low gestational age neonates frequently need mechanical ventilation for treatment of respiratory distress syndrome. BPD is a form of chronic lung disease that occurs in these patients with high incidence and is characterized by abnormal lung compliance, increased pCO_2_, lung inflammation and pulmonary hypertension following disrupted microvascular maturation and poor alveolarization. Triggers are deficiency in vascular endothelial growth factor (VEGF), matrix metalloproteases and lack of antioxidants. Oxygen partial levels *in utero* are sufficiently low (20–30 mmHg) to ensure proper angiogenesis and extracellular matrix (ECM) deposition. However, pre-term birth at an early stage interrupts ongoing lung development and instant hyperoxic conditions lead to massive oxidative stress (Valencia et al., 2018).

### Heart

Increased pulmonary arterial pressure in response to chronic hypoxia results in hypertrophy of cardiac myocytes and thickenening of the cardiac muscle wall. Cardiomyocyte cell death leads to a thinning of the remaining heart muscle as well as dilation of the chamber (dilated cardiomyopathy).

Effects of intermittent hypoxia on the heart are encountered in diseases related to sleep-disordered breathing such as *OSAS* as well as in *central apnea (CA)* independent of sleep or wakefulness. OSAS is caused by cyclic upper airway obstruction during sleep and can lead to several types of cardiovascular disorders, like systemic hypertension, cardiac arrhythmias, infarction and heart failure. CA is caused by a temporary withdrawal of the respiratory drive from the brainstem and is observed in heart failure patients in form of the Cheyne-Stokes respiration (CSR) with cycles of crescendodecrescendo ventilation and apnea/hypopnea (Costanzo et al., 2015). CSR is interpreted to be caused by an increased chemosensitivity to hypoxia and hypercapnia as well as a chemoreflex delay due to an increased circulatory time (Ponikowski et al., 2001; Giannoni et al., 2019). Production of pro-inflammatory cytokines, ROS and mediators of the renin-angiotensin system (RAS) in the carotid body underly the increased chemosensitivity (Aimo et al., 2020).

Intermittent hypoxic conditions are also present in *ischemia-reperfusion injury (IRI)* and *myocardial infarction (MI).* IRI is probably one of the best-studied examples of oxidative injury following O_2_ deprivation with subsequent O_2_ toxicity. It occurs whenever O_2_ and nutrient supply is interrupted due to blood vessel occlusion or clamping. A paradoxical situation is observed upon reperfusion. Immediate reopening of the vessel is required to restore oxygen and nutrient supply to the tissues. However, depending on the time and amount of oxygen deprivation, reperfusion induces a profound and sustained oxidative stress due to the generation of an abundance of ROS. This phenomenon was not well understood until it was determined that the physiological oxidation of hypoxanthine to xanthine and uric acid by xanthine dehydrogenase is altered during ischemia. In ischemia-reperfusion, under reduced conditions, xanthine dehydrogenase is converted to xanthine oxidase producing large amounts of harmful ROS during the conversion of hypoxanthine (Wu et al., 2018).

In the cardiovascular system, hyperoxia induces a decreased heart rate and lowered cardiac output. This is due to an increased parasympathetic tone and a rise in systemic vascular resistance. The increase in vascular resistance is explained by decreased ATP release from red blood cells and reduced NO bioavailability. Prolonged hyperoxia has an impact on endothelial NO synthase (eNOS) and increased ROS converts available NO to peroxynitrite (nitrosative stress). Also, release of NO from S-nitrosothiole is inhibited.

### Nervous System

Reduced O_2_ supply to the brain (hypoxia) is counter-acted by a regulation of cerebral blood flow and vasodilatation in order to meet the metabolic demand. Further adaptations are changes in regulation of respiratory rate, metabolism, enzyme activities and gene expression, which provide a certain hypoxic-ischemic tolerance. This tolerance can be extended to some extent with non-injurious hypoxic exposures at an appropriate time interval and dosage (“preconditioning”). However, severe hypoxia can lead to cognitive impairment and seizures. An example for an associated pathology is *acute mountain sickness*, that goes along with symptoms like headache, tiredness, dizziness and vomiting. According to the alveolar gas equation the partial pressure of oxygen in the alveoli is reduced due to a reduced atmospheric pressure at high altitudes.

Intermittent hypoxia has an enhancing effect on the sympathetic activity, carotid body activity, inflammation, oxidative stress and endothelial dysfunction. Further, the bioavailability of NO is reduced, all of which, depending on the severity of intermittent hypoxia, can contribute to an increased risk of cognitive decline and stroke.

Breathing gas with high O_2_ concentrations (hyperoxia) bears the risk of cerebral toxicity. The most dramatical manifestation are generalized tonic-clonic grand mal seizures upon exposure to pure oxygen under supra-atmospheric pressures (diving, hyperbaric chamber), named the “Paul-Bert effect”. Hyperbaric oxygenation (HBO) therapy is implicated in carbon monoxide (CO)-poisoning, decompression injury and gas embolism.

### Kidneys

The kidneys have a relatively low innate O_2_ tension also in the healthy individual, that is explained by the O_2_ shunt between the arterial and venous vessels running parallel in the kidney. *Chronic kidney disease (CKD)* frequently develops from clinical conditions like diabetes mellitus, hypertension or glomerulonephritis. Risk factors are sleep apnea syndrome and chronic obstructive pulmonary disease and progression of CKD is accelerated by increase of hypoxia in the kidneys. Hypoxia induces activity of HIF, which upregulates down-stream genes like VEGF and erythropoietin (EPO). HIF is inactivated by prolyl-hydroxylases (PHD) under normal oxygen conditions. However, loss of PHDs results in polycythemia, which is caused by EPO overproduction. Renal ischemia-reperfusion injury (IRI) can be a complication of major surgery resulting in *acute kidney injury (AKI)*, that frequently further progresses to CKD. Intermittent hypoxia as hallmark for instance of OSA can aggravate renal disease by vascular/endothelial dysfunction, oxidative stress, inflammation, increased sympathetic nervous system activity and dysregulation of the RAS, especially activation of angiotensin II. Supplemental O_2_ can alleviate hypoxic conditions also in the kidneys, but excess O_2_ also exerts toxicity in this organ which can ultimately lead to kidney disease or failure.

### Vascular System

Organ and cellular homeostasis are maintained by the vascular system, that responds to changes in O_2_. In contrast to the pulmonary circulation, the systemic circulation responds to hypoxia with vascular dilation and only with more severe hypoxia with peripheral vasoconstriction to redistribute the oxygenated blood to the organs. Chronic intermittent hypoxia (CIH) can lead to hypertension due to activation of the sympathetic nervous system and the RAS, scavenging of NO, increased levels of endothelin and altered baroflex control. A hallmark of CIH is endothelial dysfunction and oxidative stress and development of artherosclerosis or vascular disease. Hyperoxia and the associated generation of ROS reduces the bioavailability of NO resulting in an increased systemic vascular resistance.

### Eye

Prematurely born infants who receive neonatal intensive care including high O_2_ therapy to support respiration are especially prone to develop *retinopathy of prematurity (ROP).* Retinal vascularization is a process that normally develops under low O_2_ tension. Excess O_2_ leads to disorganized growth of retinal vessels, scar formation and retinal detachment, in the worst case leading to blindness (Hellstrom et al., 2013).

## Therapeutic Strategies: Where Do We Stand and What Is the Future?

Literature on outcome studies regarding the liberal use of supplemental O_2_ and consecutive hyperoxemia is heterogenous. A recent review of 37 published studies revealed that in half of the studies, statistical evaluation resulted in no detectable association between hyperoxemia and detrimental clinical outcome. Other studies, however, found higher mortality in the context of stroke, cardiac arrest and traumatic brain injury (TBI) (Stolmeijer et al., 2018). A recent meta-analysis of high-quality data from 25 randomized controlled trials including more than 16,000 patients concluded that liberal oxygen therapy (SpO_2_ = 94–99%) is very likely detrimental with regard to short-term and long-term mortality when compared to conservative administration of O_2_ (Chu et al., 2018). This overarching analysis included patients with critical illness, stroke, trauma, sepsis, cardiac arrest and myocardial infarction. However, to approach guidelines, which provide the best possible outcome, it is necessary to look into detail of the individual conditions (Selected disease-related clinic trials are listed in Table 1).

**TABLE 1:** Selected clinical trials and studies investigating outcome under oxygen therapy.

**Medical condition**	**Study/Trial**	**References**	**Outcome**
*Acute Respiratory Distress Syndrome (ARDS)*	Data analysis from 10 RCTs	Aggarwal et al., 2018 (ARDS Network)	FiO2 > 0.5; PaO_2_ > 80 mmHg: increase of mortality in a dose-dependent manner
	Multicenter RCT	Barrot et al., 2020	Early exposure to conservative oxygenation strategy (paO_2_ = 55-70 mmHg) versus liberal oxygen therapy (paO_2_ = 90-105 mmHg) did not increase survival at 28 days
	Secondary analysis of LUNG SAFE study	Madotto et al., 2020	No relationship between hyperoxemia and outcome
*Chronic Obstructive Pulmonary Disease (COPD)*	LOTT	Long-Term Oxygen Treatment Trial Research Group et al., 2016	No benefit for patients with stable COPD und moderate desaturations (mortality, time to hospitalization)
	RCT	Austin et al., 2010	Patients with acute excacerbations of COPD: titrated oxygen reduced mortality, hypercapnia and respiratory acidosis
*Myocardial Infarction (STEMI)*	Cochrane Report	Cabello et al., 2013	Need for better RCTs
	AVOID	Stub et al., 2015	Supplemental oxygen in patients with STEMI: no change in cardiac troponin, larger infarct size
	SOCCER	Khoshnood, 2018	No benefit for normoxic STEMI patients; No change in infarct size, morbidity, mortality
	DETO2X Meta-analysis from 8 RCTs	Hofmann et al., 2017; Hofmann et al., 2018; Sepehrvand et al., 2018	Suppl. Oxygen in normoxemic patients with STEMI does not reduce 1 year all cause mortality, STEMI-PCI: no significant effect on rehospitalization with MI, cardiogenic shock, stent thrombosis; No clinical benefit of supplemental O_2_ therapy
*Cardiac Arrest-Cardio-pulmonary Resuscitation (CA/CPR)*	Multicenter study	Kilgannon et al., 2010	Strong hyperoxemia (PaO_2_ > 300mmHg): increased in-hospital mortality
*Colorectal surgery*	PROXI	Meyhoff et al., 2009	Slightly earlier reoccurrence of cancer, increased mortality
	Meta-analysis of SSI studies	Podolyak et al., 2016	Perioperative supplemental oxygen does increase long-term mortality of colorectal surgery patients
	Meta-analysis of PROXI	Fonnes et al., 2016	Perioperative hyperoxia increased long-term risk of myocardial infarction and cardiovascular diseases
*Sepsis/Septic Shock*	HYPER2S multi-center RCT	Asfar et al., 2017	Adverse outcome in mechanically ventilated ICU patients; In patients with septic shock arterial hyperoxia increases risk of mortality
*Stroke Traumatic brain injury*	SOS RCT TRAUMOX1	Roffe et al., 2017; Baekgaard et al., 2019	No benefit of supplemental oxygen in patients with acute stroke; Recommends well designed conservative oxygen therapy
	RCT	Taher et al., 2016	Better outcome with liberal oxygen therapy

### Use of Oxygen During General Surgery

Perioperatively, a FiO_2_ of 0.8 vs 0.3 during anesthesia and for several hours after is mostly applied in order to prevent SSI, nausea and vomiting, and to promote wound healing. Preoxygenation with 100% O_2_ is often used in order to provide enough time for endotracheal intubation (“safe time” during apnea); however, lower concentrations of O_2_ might be preferable in order to avoid formation of atelectasis.

In the 1980s, it was proposed that giving high fractions of O_2_ in the perioperative phase might have a positive side-effect: increased ROS has bactericidal properties like an antibiotic and might therefore reduce SSI, especially in abdominal surgeries (Knighton et al., 1984). Also, oxidative bursts of neutrophils require molecular O_2_. In the following years, several further approaches to explain this beneficial effect were taken: a reduction of inflammation and a boost of local macrophages, as well as a decreased release of TNFα by leukocytes was discussed. However, a higher mortality and tumor recurrence was observed in cancer patients. Three trials reported statistically significant results (Greif et al., 2000; Belda et al., 2005; Myles et al., 2007). All other trials either did not reach statistical significance or were for other reasons inconclusive (Meyhoff, 2019). A meta-analysis from two trials on surgical site infections in patients undergoing elective colectomy did not reveal an increase in long-term mortality (Podolyak et al., 2016). The WHO guideline from 2016 advocating the use of 80% O_2_ in anesthesized, intubated and ventilated patients during and up to 6h after surgery was based on a subgroup analysis from precendent trials and initially ignored potential harmful effects, which were the main points of subsequent criticism (Meyhoff et al., 2017). Potential hazards would be an increase in mortality and respiratory complications (Meyhoff et al., 2012; Staehr-Rye et al., 2017). An earlier Cochrane review from 2015 stated that evidence was insufficient to support the use of increased perioperative O_2_ (Wetterslev et al., 2015). Therefore, the WHO re-assessed the available evidence regarding the effectiveness of high FiO_2_ to reduce SSI, and taking into consideration possible associated adverse events, the strength of the recommendation was revised from strong to conditional in 2018.

Another very recently published paper still advocates hyperoxia in noncritically ill intubated adult surgical patients (Weenink et al., 2020). The unclear risk-benefit ratio led Meyhoff and others to stress the need of large trials to obtain the required statistical power to evaluate the effects on SSIs (Meyhoff et al., 2011; Cohen et al., 2018; Meyhoff, 2019). Conflicting conclusions exist from studies comparing longterm mortality among colorectal cancer surgery patients who received 80% vs. 30% O_2_ during surgery. The PROXI trial identified a slightly earlier reoccurrence of cancer and increased mortality among cancer patients in the 80% O_2_ group (Meyhoff et al., 2009). Fonnes et al. re-analyzed data from the PROXI trial and concluded that perioperative hyperoxia might increase the long-term risk of myocardial infarction and cardiovascular diseases (Fonnes et al., 2016).

Guidelines from international medical societies differ to some extent in their specific recommendation, but caution the use of too liberal use of oxygen in the clinical routine (Mellin-Olsen et al., 2017; O’Driscoll et al., 2017).

### Oxygen in Critical Care

#### Emergency Department and Intensive Care Unit (ED/ICU)

This group of observations certainly draws upon a very heterogenous patient population with various illnesses. However, supplemental oxygen is often a first-choice treatment in the ED, and therefore the related outcome statistics are of interest. When comparing conservative (SpO_2_ = 94–98%) with conventional (SpO_2_ = 97–100%) oxygen therapy in the ICU, a significant increase in death during the ICU stay was observed in the latter group (Girardis et al., 2016). Accordingly, de Jonge et al. found an association between higher arterial partial oxygen pressures and mortality (de Jonge et al., 2008). Page et al. investigated the impact of early hyperoxia upon intubation in the emergency department (ED) in an observational study among patients who then experience normoxia later in the ICU (Page et al., 2018). The median FiO_2_ was 70%, representing a liberal use of oxygen. Their final conclusion was that hyperoxia is an independent predictor of hospital mortality, even under the relatively brief exposure time in the ED.

#### Acute Respiratory Distress Syndrome (ARDS)

According to the Berlin Definition there are three stages of ARDS severity, depending on the degree of hypoxemia leading to respiratory failure: mild (PaO_2_/FiO_2_ = 200–300 mmHg), moderate (PaO_2_/FiO_2_ = 100–200 mmHg) and severe (PaO_2_/FiO_2_ ≤ 100 mmHg). Oxygen is a priority treatment in order to restore arterial oxygenation, but oxygen toxicity on the other hand can worsen pre-existing inflammation and inflict significant lung tissue injury.

It is frequent practice to target normoxemia (PaO_2_ 85–110 mmHg) in patients with ARDS in order to provide sufficient O_2_ for avoiding hypoxemia and to prevent neurocognitive dysfunction in survivors (Mikkelsen et al., 2014). This is supported by many physicians considering a FiO_2_ below or equal 0.6 as safe and disregarding the fact that O_2_ toxicity is aggravated by lung inflammation and other co-existing pathological lung conditions. Inflammation compromises the cellular capability to properly adjust the antioxidant defense. Excessive O_2_, resulting in high PaO_2_ in ARDS patients, can lead to worsening oxygenation indices, a longer need for mechanical ventilation, longer intensive care unit and hospital stays and even to a higher in-hospital mortality (de Jonge et al., 2008; Rachmale et al., 2012; Kallet and Branson, 2016). Data analysis from 10 RCTs in ARDS Network hospitals (Clinical Research Network comprising 42 hospitals in United States; period of trials: 1996–2013) revealed that excess O_2_ (defined as FiO_2_ > 0.5; PaO_2_ > 80 mmHg) increased mortality in a dose-response manner (Aggarwal et al., 2018). Consensus recommendations of the ARDS Network therefore advise one to maintain a SaO_2_ of 88–95% at airway plateau pressures below 30 mmHg and a positive end-expiratory pressure of 5–20 mmHg.

Scientifically interested physicians are speaking for more clinical trials to investigate whether targeting mild permissive hypoxemia (PaO_2_ 55–80 mmHg; SpO_2_ 88–92%) results in improved outcomes in at-risk patients for EALI (early acute lung injury) or those with ARDS (Aggarwal and Brower, 2014). The recommendation of conservative or permissive O_2_ therapy with targets for paO_2_ of 55–80 mmHg and SpO_2_ between 88–92% were tested in several trials regarding feasibility and safety as well as benefits in outcome compared to a liberal or standard oxygen therapy.

In a before-after trial of a two-step implementation of conservative O_2_ targets in critically ill patients in 2016, Helmerhorst and colleagues could show an increase in guideline adherence for PaO_2_ targets of 55–86 mmHg and SpO_2_ of 92–95% from 47% to 68% after training and feedback of clinicians in three ICUs in the Netherlands without a significant increase in hypoxic episodes or relevant safety issues. An improved hospital survival and more ventilator free days could be shown as well in these 15.000 examined patients (Helmerhorst et al., 2016). These were patients with mechanical ventilation, but not necessarily with ARDS. As these PaO_2_ targets are poorly validated in prospective trials for ARDS patients, Barrot et al. very recently conducted a multicenter randomized open label trial on conservative (PaO_2_ = 55–70 mmHg, SpO_2_ = 88–92% for 7 days) oxygen therapy in ARDS patients in France in 13 ICUs between 2016 and 2018 (Barrot et al., 2020). This study, however, was stopped prematurely due to safety concerns after 205 patients were enrolled. The 28-day mortality in the conservative group was 34.3% compared to 26.5% in the liberal control group. After 90 days, 44.4% died in the treatment group while only 30.4% died in the conservative group. Also, four cases of mesenteric ischemia occurred in the conservative group.

In 2020, Mackle and colleagues published a similar trial (ICU-ROX) comparing a conservative oxygen therapy to standard care in mechanically ventilated ICU patients (Mackle et al., 2020). Although in both cohorts, the mean PaO_2_/FiO_2_ ratio was < 300, it was not a study explicitly conducted in ARDS patients. Primary outcome was ventilator free days and no difference could be demonstrated in 1000 randomized patients. A sub-study from the epidemiological LUNG SAFE study (from 2016) assessed the association of hyperoxemia (on admission or sustained) and excessive oxygen use in early ARDS. In this cohort study, no relationship could be found to worse outcome in regard to hypoxemia and high FiO_2_ (Madotto et al., 2020). Generally, hyperoxia (and mild hyperoxia) aggravate and synergistically worsen VILI phenomenons such as an increase in pulmonary permeability and edema formation, decreased surfactant and lung compliance, and an increase in inflammatory markers and apoptosis (Kallet and Branson, 2016) (and references therein).

#### Chronic Obstructive Pulmonary Disease (COPD)

High O_2_ concentrations might lead to hypercapnia (due to an oxygen-induced ventilation/perfusion mismatch and the Haldane effect) in patients with exacerbations of chronic obstructive pulmonary disease (AECOPD) and might increase mortality, which is why a target SpO_2_ of 88–92% is recommended in these patients. According to the LOTT trial, long-term supplemental O_2_ offered no benefit for patients with stable COPD and moderate desaturations with regard to mortality or time to first hospitalization (Long-Term Oxygen Treatment Trial Research Group et al., 2016). An Australian trial which investigated the effect of titrated oxygen (SpO_2_: 88–92%) versus high-flow O_2_ without SpO_2_ target in the prehospital period in patients with presumed acute excacerbation of COPD, revealed a significantly reduced risk of death (Austin et al., 2010).

#### Cardiac Arrest and Cardiopulmonary Resuscitation (CA/CPR)

Cardiac arrest and cardiopulmonary resuscitation are situations that exemplify ischemia-reperfusion injury, which has proven to be aggravated by excess supplemental oxygen. Additionally, hyperoxia leads to vasoconstriction in the cardiovascular system. Experimental evidence would clearly imply that excess oxygen is contra-indicated in this context. Also, some clinical trials showed increased mortality among patients receiving high levels of supplemental O_2_. Although a couple of observational studies could not link hyperoxemia (during and 24 h after CPR) to adverse outcome, some larger studies implicated a dose-dependent relationship between oxygen and in-hospital mortality. Strong hyperoxemia (PaO_2_ over 300 mmHg) was clearly linked to increased in-hospital mortality (Kilgannon et al., 2010). Also, hyperoxemia might result in an increased risk of poor neurological outcome (Roberts et al., 2018). Current guidelines therefore recommend a titrated and individually tailored O_2_ therapy (target: SpO_2_ = 94–98%) instead of the previous liberal O_2_ therapy upon return of spontaneous circulation (ROSC).

#### ST-Elevation Myocardial Infarction (STEMI/MI)

After publication of conflicting data from several trials, a Cochrane report from 2013 indicated the need for better randomized controlled trials (RCTs) concerning the role of supplemental O_2_ for patients with suspected myocardial infarction (Cabello et al., 2013). Consequently, the AVOID study reported no change in cardiac Troponin, but a larger infarct size as revealed in cardiac magnetic resonance imaging (CMRI) in patients with STEMI who were treated with supplemental oxygen (Stub et al., 2015). In the SOCCER study, normoxic STEMI patients, or patients with suspected MI, have been shown to neither benefit from supplemental O_2_ therapy, nor have negative effects with regard to infarct size, morbidity and mortality (Khoshnood, 2018). Similiar conclusions were drawn in the DETO2X trial (Hofmann et al., 2017, 2018; Sepehrvand et al., 2018). Following the results from these studies, it is advisable to reconsider guidelines, obmitting supplemental oxygen therapy in normoxic STEMI patients and only providing oxygen to those patients who have low blood O_2_ saturation (Khoshnood, 2018). The *European Society of Cardiology (ESC)* guideline from 2017 recommends against the routine adminstration of supplemental oxygen in patients with MI, when peripheral oxygen saturation is more than 90% (Ibanez et al., 2018). Following the results from these studies, it is advisable to reconsider guidelines, obmitting supplemental O_2_ therapy in normoxic STEMI patients and only providing O_2_ to those patients who have low blood O_2_ saturation (Khoshnood, 2018). The *European Society of Cardiology (ESC)* guideline from 2017 recommends against the routine adminstration of supplemental O_2_ in patients with MI, when peripheral O_2_ saturation is more than 90% (Ibanez et al., 2018).

#### Heart Failure (HF)

A meta-analysis revealed that heart failure patients are most susceptible to hyperoxia-induced hemodynamic changes (Smit et al., 2018). Oxygen therapy is one possible approach for treating HF-patients with Cheyne-Stokes respiration beside continuous positive airway pressure (CPAP), dynamic CO_2_ administration and pharmacotherapies (Borrelli et al., 2017). Oxygen reduces the response of peripheral chemoreceptors and increases the arterial level of CO_2_. Several studies have shown a decrease in the apnea-hypopnea index (AHI), a decrease in sympathetic activity and an improvement of cardiac function. As key pathological mechanisms in HF syndrome are oxidative stress and inflammation, adjunctive therapies with antioxidants have been proposed and tested, however, with very limited success (Aimo et al., 2020).

#### Sepsis and Septic Shock

Sepsis is a dysregulated host-response to infection that might lead to life-threatening organ dysfunction. While priority treatment is focused on anti-microbial therapy, O_2_ supplementation requires further attention. Oxygen might enhance antimicrobial host defense (as is argued in the case of SSI) and act as a vasoconstrictor, which stabilizes the hemodynamics of shock patients, but it might also reduce O_2_ uptake. Sepsis patients frequently exhibit higher venous oxygen levels (ScvO_2_) due to mitochondrial dysfunction and reduced oxygen extraction, which is linked to increased mortality. The HYPER2S study, a multicenter randomized clinical trial, reported adverse outcomes associated with hyperoxaemia (100% vs. titrated O_2_ therapy) in mechanically ventilated ICU patients (Asfar et al., 2017).

In *ischemic brain injury* (e.g., as in stroke and intracranical bleeding), high FiO_2_ has proven to be detrimental. PaO_2_ values higher than 170–200 mmHg have been shown to induce cerebral ischemia and poor neurological outcome, and still higher FiO_2_s (more than 300 mmHg) have even increased mortality in clinical trials.

#### Stroke

Oxygen is a double-edged sword, especially in neurological conditions. It has a positive impact on hemodynamics and might improve clinical deficits, but the generated ROS might lead to further neuronal damage. Also, in this group of patients, study results are ambivalent. With regard to therapeutic effects, a large clinical trial with 8003 patients did not reveal any benefit derived from supplemental oxygen therapy in patients with acute stroke (Roffe et al., 2017).

#### Subarachnoid Hemorrhage (SAH)

Despite the requirement of oxygenation, a recent observational study revealed a strong link between hyperoxemia (PaO_2_ > 120 mmHg) and adverse outcome (Yokoyama et al., 2018).

#### Traumatic Brain Injury (TBI)

Ischemic injury after TBI can be counteracted by supplemental oxygen, thereby improving brain oxygenation. A study from 2016 offered evidence that TBI patients treated with restricted oxygen suffered from a higher degree of disability 6 months after the incidents than patients provided with liberal oxygen (Taher et al., 2016). Other studies established a link between higher oxygen partial pressures and increased mortality after TBI. Against this background, recent studies indicate that a well designed conservative oxygen therapy is both feasible and advisable (Baekgaard et al., 2019).

### Antioxidant Therapies

Oxidative stress is a hallmark of many diseases, let it be neurological, cardiovascular or pulmonary. As the innate antioxidative molecules are reasonably well understood, it seems obvious to use antioxidative drugs or enzymes to treat such conditions. However, clinical success in many cases was far below expectations. A reason might be, that the redox systems underlie multiple influencing factors and interference at one side might disrupt other processes. Excessive ROS ist certainly detrimental, but ROS are also important cellular messengers. Also, the antioxidative mechanisms are frequently cell-type specific, why a “one-fits-all” therapy might not be appropriate.

Administration of antioxidants via the oral, intratracheal or intravenous route have been tested to treat for instance pulmonary oxidant stress (Christofidou-Solomidou and Muzykantov, 2006). Recombinant human SOD has been shown to be protective in premature infants exposed to hyperoxia. In order to deliver these drugs to their target more efficiently several strategies are developed including liposomal delivery and coupling to antibodies. Oxidative stress also plays an important role in hypertension, where antioxidative treatments have been shown to be effective in animal models, but these results have been proven difficult to be translated into the treatment of the human disease (Sorriento et al., 2018). Reasons might be the inhomogenous study populations with regard to co-morbidities, interactions with other medications or simply substance-specific properties with regard to delivery and half-life. Vitamin D supplementation as well as antihypertensive drugs with antioxidant properties like carvediol, celiprolol and nebivolol are currently used and further investigated. Antioxidative treatment in HF comprised vitamin E, vitamin C, the mitochondrial antioxidants elamipretide and coenzyme Q. Several trials were designed to test the efficiency, but yielded either no or slightly negative effects on cardiovascular outcome in the case of vitamin E. Further trials warrant to test possibly positive effects of vitamin C, coenzyme Q and elamipretide (Aimo et al., 2020).

## How Much Oxygen Is Safe? Conclusion From Animal Experiments

We still do not have a clear perception of how much O_2_ can be regarded as “safe” in humans. Oxygen toxicity increases with concentration, prolonged exposure and concomitant “second hit” adverse conditions such as lung inflammation, sepsis or mechanical ventilation with high tidal volumes. An immediate instinct would be to pursue animal experiments to test for a safe therapeutic range under different O_2_ conditions. However, the susceptibility to O_2_ toxicity varies considerably among animal species and is further dependent upon age and strain. Small animals, especially rodents, are usually the first choice for testing therapeutics. Some significant conclusions could be drawn from mouse, rat or rabbit experiments. However, the susceptibility of different mouse strains to hyperoxic lung injury and O_2_ toxicity in general varies considerably. A neonatal mouse is at a developmental lung stage similar to a 25-26 week old infant (Zoetis and Hurtt, 2003) and is therefore frequently used as a rodent model of human BPD. Genome-wide analysis has revealed mouse strain-dependent and -independent patterns of gene expression that are involved in the maturation of the murine lungs (Tiono et al., 2019). Also, the antioxidative and inflammatory response has proven to be strain-dependent, why some researchers argue to use out-bred animals instead of in-bred strains. Usefulness and limitations of animal models are well recognized as an example in studying retinopathy of prematurity. ROP models have been developed in kitten, mouse and rat (Madan and Penn, 2003). While kittens are excellent models to study neovascularization, the pathogenesis is not like in humans. Mice are similar, but at least offer the option of transgenic technology. Rats’ pathogenesis most closely mimics the human condition, but rats cannot be manipulated so easily as mice. Even if an animal displays similar mechanisms as observed in humans, experimenters in most cases have to use healthy animals, which have a different sensitivity to oxygen as a premature infant with lung disease. Also oxygen exposure might be more extreme and therefore not really relevant for the clinical setting.

However, because of the abovementioned reasons, studies in small animals might help to investigate basic mechanisms, but are not directly translateable to human conditions. In general, primates are closer to humans and show similiar responses to O_2_, but they are not always available. Oxygen toxicity has been investigated in primates in order to better understand the human condition (Robinson et al., 1974). And still, the genetic predisposition, age and co-morbidities are factors, that differ between human individuals. Increasing age, for instance, goes along with oxidative processes (“oxidative stress theory of aging”), which have an impact on protein levels and function as well as on physiology (Lin and Beal, 2003). Co-morbidities vary between patients and in many of the prevalent disease conditions (cardiovascular diseases, neurodegeneration, diabetes, etc.) is oxidative stress a confounder, which might change the patient’s susceptibility to oxygen-related injury as well as the response to antioxidant therapy. The approach of personalized medicine will help to adjust pharmacological treatments to the individual’s needs, but is somewhat still in its infancy due to limitations in cost and also in scientific progress.

## Conclusion and Perspective

The therapeutical window for supplemental O_2_ (between avoiding hypoxemia and hyperoxemia) is ill defined, partly because it is ultimately dependent upon the individual, his current co-morbidities and adverse conditions, and probably because the consequences of “too much O_2_” are not immediately recognizeable. Also, of consequence is that clinical studies are heterogenous with respect to design and results. Experimental options are limited, as conclusions from animal and cell culture experiments are not easily translateable into clinical practice. More recent guidelines tend to recommend a conservative O_2_ therapy with lower targets of blood oxygenation. At present, novel devices are being developed that aim to better tailor individual O_2_ therapy by measuring blood oxygenation and adjusting the ventilator and the inspiratory oxygen fraction. This would serve to better maintain oxygen homeostasis in the human body and to avoid long and excessive episodes of potentially harmful hyperoxia.

## Author Contributions

All authors contributed to the contents of the manuscript. VT wrote the first draft and all authors contributed to manuscript revision, read and approved the submitted version.

## Conflict of Interest

The authors declare that the research was conducted in the absence of any commercial or financial relationships that could be construed as a potential conflict of interest.

## Abbreviations

FiO_2_: fraction of inspired oxygen
ICU: intensive care unit
paO_2_: arterial partial pressure of oxygen
PCI: percutaneous coronary intervention
RCT: randomized controlled trial
SSI: surgical site infection
STEMI: ST-elevation myocardial infarction.
